# Immune mobilising T cell receptors redirect polyclonal CD8^+^ T cells in chronic HIV infection to form immunological synapses

**DOI:** 10.1038/s41598-022-23228-3

**Published:** 2022-11-01

**Authors:** Zoë Wallace, Jakub Kopycinski, Hongbing Yang, Michelle L. McCully, Christian Eggeling, Jakub Chojnacki, Lucy Dorrell

**Affiliations:** 1 Nuffield Department of Medicine, University of Oxford, Oxford, UK; 2 Oxford NIHR Biomedical Research Centre, University of Oxford, Oxford, UK; 3 Immunocore Ltd, 92 Park Drive, Abingdon, Oxfordshire, UK; 4 MRC Human Immunology Unit, Weatherall Institute of Molecular Medicine, University of Oxford, Oxford, UK; 5 Leibniz Institute of Photonic Technology & Institute of Applied Optics and Biophysics, Friedrich-Schiller University, Jena, Germany

**Keywords:** Cellular imaging, Infectious diseases, HIV infections

## Abstract

T cell exhaustion develops in human immunodeficiency virus (HIV) infection due to chronic viral antigenic stimulation. This adaptive response primarily affects virus-specific CD8^+^ T cells, which may remain dysfunctional despite viral load-reducing antiretroviral therapy; however, abnormalities may also be evident in non-HIV-specific populations. Both could limit the efficacy of cell therapies against viral reservoirs. Here, we show that bulk (polyclonal) CD8^+^ T cells from people living with HIV (PLWH) express proposed markers of dysfunctional HIV-specific T cells at high levels yet form lytic immunological synapses (IS) and eliminate primary resting infected (HIV Gag^lo^) CD4^+^ T cells, when redirected by potent bispecific T cell-retargeting molecules, Immune mobilising monoclonal T cell receptors (TCR) Against Virus (ImmTAV). While PLWH CD8^+^ T cells are functionally impaired when compared to CD8^+^ T cells from HIV-naïve donors, ImmTAV redirection enables them to eliminate Gag^lo^ CD4^+^ T cells that are insensitive to autologous HIV-specific cytolytic T cells. ImmTAV molecules may therefore be able to target HIV reservoirs, which represent a major barrier to a cure.

## Introduction

During the early stages of HIV infection, CD8^+^ T cells are effective at limiting viral replication. However, as a consequence of chronic antigenic stimulation and the resulting pro-inflammatory environment, both CD8^+^ and CD4^+^ T cells develop functional defects, including impaired proliferation, cytotoxicity and IL-2 production^1,2^. Dysfunctional T cells are typically marked by the persistent expression of inhibitory receptors. For example, high PD-1 expression on virus-specific CD8^+^ T cells is linked to impaired functionality in untreated HIV infection, although it is to some extent reversible with antiretroviral therapy (ART)^3,4^. Upregulation of other inhibitory receptors, including LAG3, TIM3, TIGIT, 2B4 and CD160, individually or in combination, has also been associated with CD8^+^ T cell exhaustion and disease progression^5,6^. Recent studies have identified two memory progenitor subsets expressing the transcription factor TCF-1: a PD-1^+^TIGIT^+^ population that differentiates into dysfunctional, terminally differentiated CD8^+^ T cells upon antigenic stimulation, and a PD-1^-^TIGIT^-^ stem memory population that gives rise to functional memory CD8^+^ T cells^7–9^. The discovery of these progenitors has shed light on the mechanisms driving clinical responses to immune checkpoint blockade and therapeutic vaccination. Given the persistence of dysfunctional virus-specific CD8^+^ T cells during long-term ART, these findings could have implications for immunotherapeutic approaches to elimination of HIV reservoirs.

A key contributing factor to the persistence of HIV reservoirs during ART is the lack of sufficient viral peptide presentation by infected CD4^+^ T cells to trigger cytotoxic T lymphocyte (CTL) killing. A proportion of HIV-infected CD4^+^ T cells in ART-treated individuals are transcriptionally active and may express viral proteins such as Gag even in the absence of new virion production, although expression is often very low^10–13^. These Gag^+^ cells may be susceptible to immune clearance by CTL, as suggested by observations in HIV controllers^14^. However, new approaches are needed to aid detection and clearance of HIV-infected cells in the majority of people living with HIV (PLWH). T cell retargeting therapies are an attractive approach for the elimination of HIV reservoirs as they are designed to circumvent ineffective or exhausted antigen-specific T cell responses.

ImmTAX (Immune mobilising monoclonal TCRs Against X disease) molecules are a class of bispecific T cell engager, comprising a soluble affinity-enhanced T cell receptor (TCR) coupled to an anti-CD3 single chain variable fragment (scFv)^15–20^. In the case of ImmTAV molecules, the TCR binds to viral antigen-derived peptide-HLA-I (pHLA) complexes with a stronger affinity than that of a natural TCR by approximately one million-fold, enabling detection of virus-infected cells that normally evade natural host immune responses due to low pHLA copy number on the cell surface. The micromolar affinity anti-CD3 scFv recruits and activates polyclonal T cells, leading to the lysis of the target cell through delivery of pro-apoptotic molecules. The weaker affinity of this anti-CD3 domain permits serial engagement of CD8^+^ T cells regardless of their native antigen specificity, enabling recruitment of many more effector cells than are typically activated in the context of natural infection or therapeutic vaccination. ImmTAV molecules therefore function independently of HIV-specific CD8^+^ T cells that may be slow to re-expand and execute effector functions upon activation^6^.

We previously reported that ex vivo CD4^+^ T cells from PLWH treated with antiretroviral therapy were susceptible to ImmTAV-mediated elimination by HIV-naïve donor CD8^+^ T cells^17^. At picomolar concentrations and at low effector/target cell ratios, these Gag-specific molecules mediated highly efficient elimination of primary CD4^+^ T cells upon in vitro reactivation of integrated HIV. However, in this study autologous bulk CD8^+^ T cells from PLWH were not as efficient as HIV-naïve donor CD8^+^ T cells when used as effector cells, suggesting an impact of chronic HIV infection on the functionality of non-HIV-specific CD8^+^ T cells that has not been fully appreciated to date.

In this study, we sought to identify the potential mechanisms underlying the differences between HIV-naïve donor and ART-treated PLWH T cells by assessing the latter for signs of exhaustion, as well as their ability to form functional immune synapses (IS) upon ImmTAV redirection. Engagement of the TCR with cognate pHLA triggers the formation of TCR microclusters, followed by a signalling cascade that results in cytoskeletal rearrangements, cytolytic granule convergence and microtubule organising centre (MTOC) polarisation to the IS^21,22^. Release of perforin and cytolytic granules into the synaptic cleft results in killing of the target cell^23,24^. As effective CD8^+^ T cell killing depends on the coordination of these processes, delayed and/or disorganised IS formation may result in reduced killing capacity^25,26^. The quality of IS mediated by tumour-specific chimeric antigen receptor T cells (CAR-T) in vitro was predictive of the efficacy of CAR-T cell therapy in an in vivo xenograft model, highlighting the utility of IS analysis^27^. We extended our previous findings by evaluating whether Gag-specific ImmTAV molecules could efficiently redirect PLWH T cells towards resting infected CD4^+^ T cells, as a model of transcriptionally active cells that make up a substantial component of HIV reservoirs in ART-suppressed individuals^28^. Here we found that CD8^+^ T cells from ART-treated PLWH showed elevated TIGIT expression and formed poorly synchronised IS but when re-directed by an HIV-specific ImmTAV molecule were able to eliminate resting infected primary CD4^+^ T cells that expressed low levels of Gag proteins.

## Results

### CD8^+^ T cells from chronic HIV infected subjects degranulate normally upon activation despite being enriched for TIGIT expression

Previously we showed that ImmTAV-mediated killing of HIV-infected CD4^+^ T cells by polyclonal CD8^+^ T cells from ART-treated PLWH required a tenfold higher effector to target ratio to achieve an equivalent effect to CD8^+^ T cells from HIV-naïve donors, highlighting a difference in their functional capacity (Supplementary Table S1^17^). T cell exhaustion has been linked to the upregulation in HIV-specific CTL of co-inhibitory receptors such as PD-1, and the ecto-enzyme CD39. As CD39 has been associated with T cell exhaustion in bulk CD8^+^ T cells or virus-specific CTLs from donors with chronic viral infections (but not CD8^+^ T cells or latent virus-specific CTLs from healthy donors) we compared the expression of PD-1 and CD39 in bulk CD8^+^ T cell populations from PLWH who were durably suppressed on ART (Supplementary Table S2) with those of HIV-naïve donors^3,29,30^.

**Figure 1:**
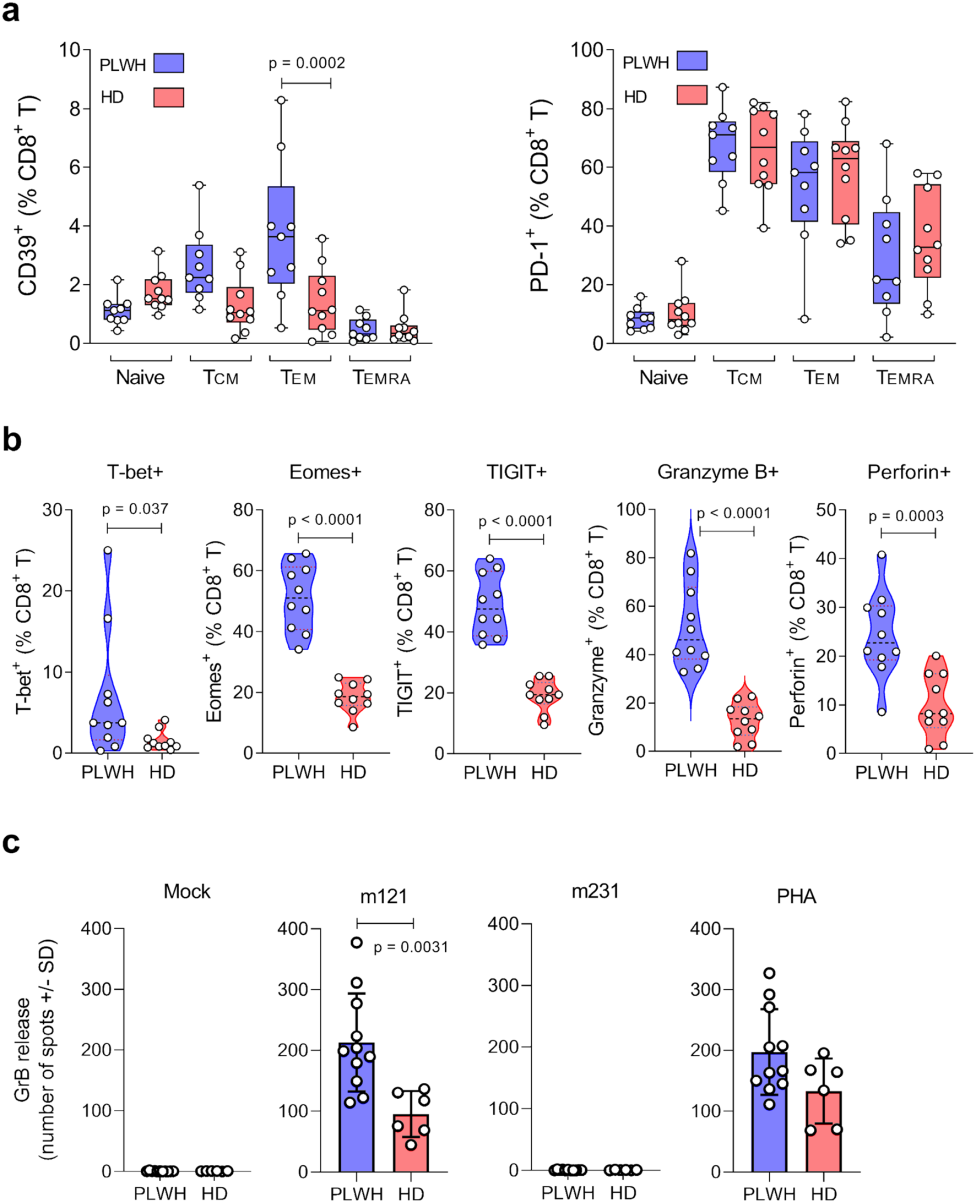
Phenotypic analysis of CD8^+^ T cells from people living with HIV and HIV-naïve donors. CD8^+^ T cells from a total of 26 virologically suppressed PLWH (PLWH, blue) and ten HIV-naïve donors (HD, red) were analysed for phenotype and/or production of granzyme B. (**a**) CD39 and PD-1 in naïve, T_CM_, T_EM_ and T_EMRA_ subpopulations and plotted as percentage positive of gated (PLWH, n = 10; HD, n = 10). (**b**) T-bet, Eomes, TIGIT, granzyme B and perforin in the CD3^+^CD8^+^ population (ex vivo) and plotted as percentage positive in each population (PLWH, n = 9; HD, n = 10). (**c**) Granzyme B-producing cells in ex vivo PBMC (PLWH, n = 11; HD, n = 6) were quantified by ELISpot after culture in triplicate with SL9 peptide-pulsed T2 cells for 48 h (E:T = 1:1), together with m121 ImmTAV (0.5 nM), a non-binding control TCR fusion protein, m231 (1 nM) or no ImmTAV (mock); PHA stimulation was used as a positive control for cell viability. Negative control wells (no TCR) yielded spot-forming cell counts < 3/well in all subjects, for both peptide-pulsed and unpulsed conditions. Results are plotted as mean ± SD for each condition. Groups were analysed by Mann–Whitney test or 1-way ANOVA with Sidak’s multiple comparisons.

These groups were comparable with respect to the proportion of naïve, effector memory (T_EM_), central memory (T_CM_) and terminally differentiated effector (T_EMRA_) cells (Supplementary Fig. S1). Furthermore, CD8^+^ T cell memory subsets from the same PLWH showed similar frequencies of cells expressing PD-1 and/or CD39 to HIV-naïve donors, with the exception of T_EM_, which were significantly enriched for CD39^+^ cells in the PLWH cohort (Fig. 1a). Extending our analysis to other markers that may be aberrantly expressed in chronic HIV infection (TIGIT and T cell transcription factors (Eomes, T-bet))^31,32^, we found a significant enrichment of TIGIT^+^ cells within total CD8^+^ T cells of PLWH when compared to HIV-naïve donors (48.9% vs. 19.1%, *p* < 0.0001). However, the PLWH also had a higher frequency of perforin^+^ and granzyme B^+^ CD8^+^ T cells (Fig. 1b). Furthermore, significantly higher frequencies of CD8^+^ T cells that were either T-bet^+^ or Eomes^+^ were present in PLWH, with a disproportionate number of Eomes^hi^ cells, consistent with prior observations by Buggert et al. (Fig. 1b; further subset phenotypic analysis in Supplementary Fig. S1)^32^.

In parallel, we explored the capacity of PLWH CD8^+^ T cells to secrete lytic granules upon activation using a 48-h granzyme B ELISpot assay^18^. Cognate peptide-pulsed (SLYNTVATL, SL9) T2 cells were used as targets, to maximise pHLA ligand availability for TCR binding, and thus reduce the potential for variability in target cell antigen expression in the assay. The frequencies of granzyme B spot-forming units were significantly higher in PLWH (mean ± SD: 95 ± 38 SFU/well for HIV-naïve donors and 213 ± 81 SFU/well for PLWH; *p* = 0.003) indicating that polyclonal CD8^+^ T cells from PLWH were not deficient in lytic granule production (Fig. 1c). These data were consistent with the higher expression of granzyme B in the flow cytometric analysis of PLWH (Fig. 1b). These observations suggested that CD8^+^ T cells from PLWH had intact lytic function despite high levels of TIGIT. This is consistent with a recent study that showed that TIGIT expression in CD8^+^ T cells correlated with cytotoxic capacity in PLWH and thus is not necessarily an indicator of exhaustion^33^.

### ImmTAV molecules enable polyclonal CD8^+^ T cells from PLWH to form lytic immune synapses

**Figure 2:**
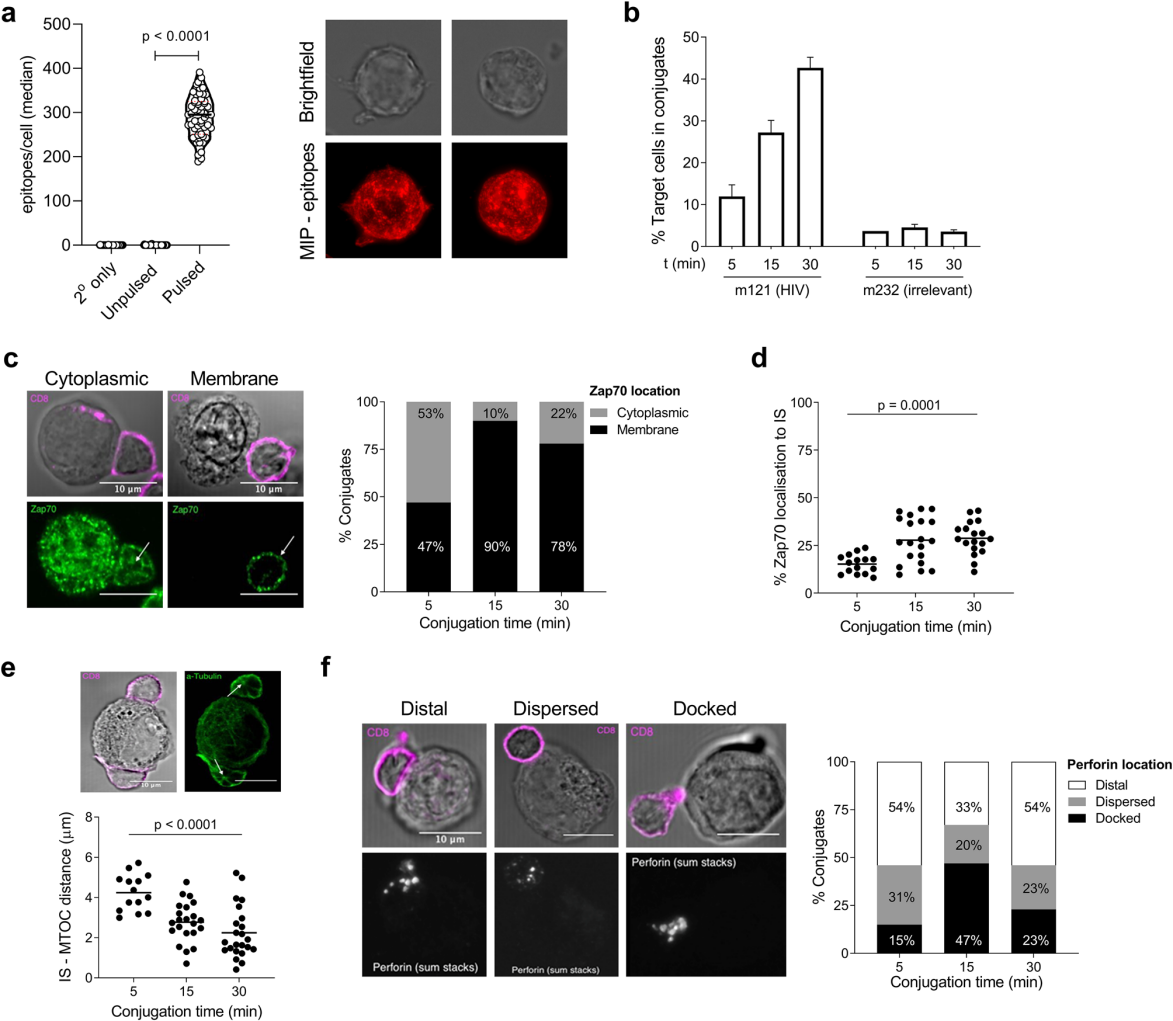
Time course of immunological synapse (IS) formation by ImmTAV-redirected HIV-naïve donor CD8^+^ T cells. SL9-peptide pulsed T2s were (**a**) stained for surface epitopes using a labelled TCR and imaged by TIRF microscopy for epitope/cell quantification (brightfield and maximum intensity projection (MIP) of labelled TCR shown; one dot represents one cell). These targets were then co-cultured with HIV-naïve donor CD8^+^ T cells (E:T of 1:1) for 5, 15 or 30 min before confocal microscopy imaging and analysis. (**b**) % of target cells in conjugates with CD8^+^ T cells over time in the presence of HIV-specific ImmTAV (m121, 0.5 nM) or irrelevant TCR-anti-CD3 fusion protein (ImmTAX, m232, 1 nM). C-F all show IS formation in the presence of the HIV ImmTAV, m121. (**c**) Representative confocal images of conjugates showing CD8^+^ T cell Zap70 (green) localisation in the cytoplasm (left) or in the plasma membrane (right). The percentage of conjugates with Zap70 localised to the cytoplasm (grey) or membrane (black) was calculated at different conjugation times. (**d**) The percentage Zap70 localised at the IS in CD8^+^ T cells. (**e**) Confocal images of α-tubulin (green) to define MTOC location (indicated with white arrow) and distance from IS to MTOC (μM). (**f**) Confocal microscopy images of perforin staining (summed Z stack shown in white) and % conjugates with distal, dispersed or docked perforin in the CD8^+^ T cells. For D & E each dot represents one T2-CD8^+^ T cell conjugate (0.5 nM m121 for **c**–**f**); 1 slide (representing 1 donor) was analysed per time-point; magenta = CD8 on differential interference contrast image (DIC). Means shown and data were analysed by one-way ANOVA.

To further probe the functionality of PLWH CD8^+^ T cells, we next assessed their capacity to form IS with peptide pulsed T2 cells when redirected by an HIV ImmTAV. Imaging of the pulsed T2 cells after staining with the same HIV TCR, in a biotinylated and fluorescently tagged format, confirmed that the target cells had a high number of peptide-HLA for recognition by the HIV ImmTAV (median 295 epitopes/cell, Fig. 2a), thus confirming their utility as ‘model antigen-presenting cells’ for investigation of ImmTAV-mediated IS formation. The experimental conditions for observation of IS by confocal microscopy were established using HIV-naïve donor CD8^+^ T cells and SL9 peptide-pulsed T2 cells, which were mixed and then imaged over a 30-min period. IS formation is initiated by the formation of a conjugate between the effector and target cell, with the interaction being stabilised by ligation of lymphocyte function-associated antigen-1 (LFA-1) and intracellular adhesion-1 (ICAM-1) on the respective cells^34^. The percentage of T2 cells in conjugates with HIV ImmTAV (m121)-redirected CD8^+^ T cells increased over time to a mean maximum of 42.7% at 30 min; by contrast, very few synapses formed in the presence of an ImmTAX molecule (‘m232’, used as an irrelevant TCR control) over the same time period (maximum 3.9% T2 cells, Fig. 2b). F-actin polarisation significantly decreased over time, consistent with the previously described cytoskeletal rearrangements that lead to clearance from the synapse (Supplementary Fig. S2)^22,35^.

Evidence of T cell activation was observed, with the redistribution of Zap70 to the cell membrane in the majority (90%) of HIV ImmTAV-redirected CD8^+^ T cells in conjugates by 15 min (Fig. 2c) and localisation at the synapse, reaching a plateau between 15 and 30 min (28.8% localised; Fig. 2d). Maximal microtubule organising centre (MTOC) polarisation to the synapse (mean 2.2 μM) was attained by 30 min (Fig. 2e) and perforin docking peaked at 15 min (47% of conjugates; Fig. 2f). Comparison of synapses formed by ImmTAV-redirected CD8^+^ T cells from three HIV-naïve donors at 15 min showed that Zap70 and MTOC polarisation were uniform between donors, whereas the proportion of conjugates with docked perforin at this stage varied from 27 to 47% (Supplementary Fig. S2). The 15-min time point was used in all subsequent investigations as it captured maximal polarisation of the synapse proteins studied, indicating synchrony in conjugate formation.

In parallel, we used live cell imaging to obtain direct evidence that IS formation results in target cell lysis. For this, we used HIV-infected Jurkat T cells as targets as their large size relative to primary CD8^+^ T cells eliminated the need for a lineage dye. Infected Jurkat T cells were efficiently killed within 30 min of co-culture, indicated by bleb formation and accumulation of cellular debris (Supplementary Fig. S3). Together, these data showed that ImmTAV molecules could be used to demonstrate immunological synapse formation between HIV antigen-positive targets and primary CD8^+^ T cells.

**Figure 3:**
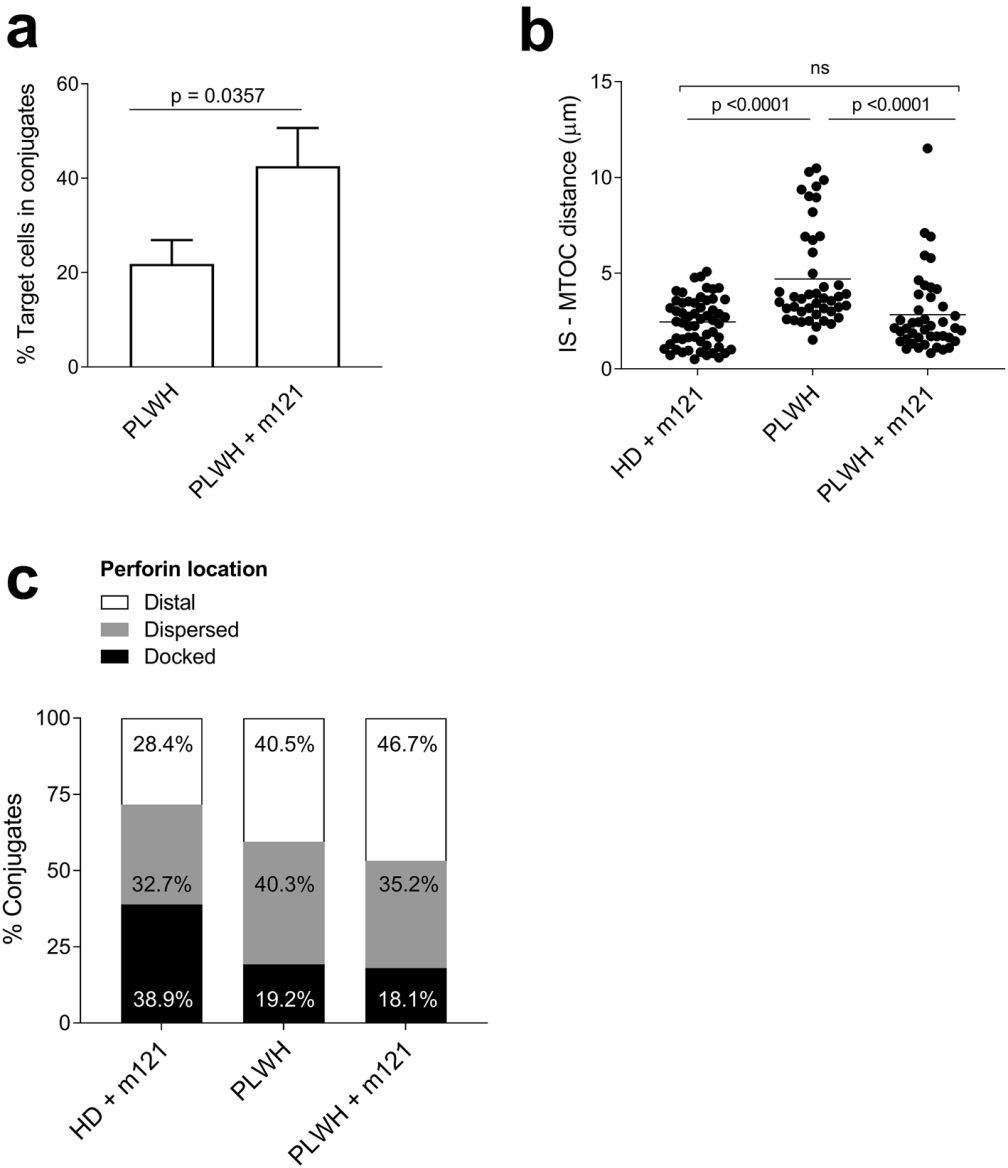
Comparison of ImmTAV-mediated immunological synapses formed by CD8^+^ T cells from HIV-naïve subjects and PLWH. SL9-pulsed T2 cells were cultured with HIV-naïve donor (HD) CD8^+^ T cells (n = 3) or SL9 peptide pre-stimulated PLWH CD8^+^ T cells (n = 3) at a ratio of 1:1 in the presence or absence of m121 ImmTAV (0.5 nM). (**a**) Quantification of conjugates formed after 15 min with pre-stimulated PLWH ± ImmTAV (n > 50 targets counted/slide, blinded analysis) and plotted as mean ± SD). (**b**) Quantification of MTOC distance from the IS (µM; data were analysed by one-way ANOVA with correction for multiple comparisons) and (**c**) % conjugates with distal, dispersed or docked expression of perforin in HD and PLWH CD8^+^ T cells. At least 10 conjugates per subject and per condition were analysed and data were pooled.

Next, we investigated the quality of IS formed by CD8^+^ T cells from PLWH subjects using the approach described for HIV-naïve donors. On initial attempts to identify CD8^+^ T cell-target conjugate pairs using CD8^+^ T cells from PLWH very few conjugates could be identified, indicating that pre-existing SL9-specific CTL were unable to form stable IS or that they were too infrequent to allow detection. This was confirmed by pre-stimulation of CD8^+^ T cells with SL9 peptide for 10 days to enrich for these cells, which resulted in the formation of more conjugates than ex vivo CD8^+^ T cells from the same individuals, but without apparent effects on mobilisation of Zap70 and MTOC and polarisation of perforin (Supplementary Fig. S4). Pre-stimulation was then used to enable a comparison of IS elicited by HIV ImmTAV molecules with IS formed by pre-existing SL9-specific CTL. ImmTAV redirection elicited a higher frequency of effector/target conjugates than did PLWH CD8^+^ T cells alone under these conditions [mean (SD) of 43% (8.1%) vs. 22% (5%) of targets (n = 3, Mann–Whitney, *p* = 0.04)] by 15 min (Fig. 3a). Importantly, when PLWH CD8^+^ T cells were redirected by the ImmTAV, MTOC polarisation was similar to that observed with HIV-naïve donor CD8^+^ T cells (n = 3, mean ± SD of 2.8 ± 2.1 and 2.4 ± 1.3 μm respectively; Fig. 3b). However, the ImmTAV had minimal effect on perforin mobilisation (Fig. 3c). By contrast, few mature ‘natural’ IS were formed by HIV-specific CTL in the absence of the HIV ImmTAV, as defined by the extent of MTOC polarisation (mean ± SD of IS-MTOC distance 4.7 ± 2.6 μm; Fig. 3b). Increasing the E:T ratio to 2:1 increased the number of conjugates formed but did not affect MTOC polarisation nor perforin mobilisation (Supplementary Fig. S5), suggesting that these subjects’ pre-existing HIV-specific CTL were dysfunctional.

Taken together, these data suggested that CD8^+^ T cells from PLWH could form functional IS upon ImmTAV redirection, although MTOC polarisation was poorly coordinated with perforin convergence. In addition, the IS analysis indicated that in the absence of the ImmTAV the same subjects’ CD8^+^ T cells, which contained SL9-specific CTL, were unable to form functional IS within the time course of the assay.

### ImmTAV molecules efficiently redirect CD8^+^ T cells from HIV-naïve donors to form lytic immune synapses with resting infected CD4^+^ T cells

**Figure 4:**
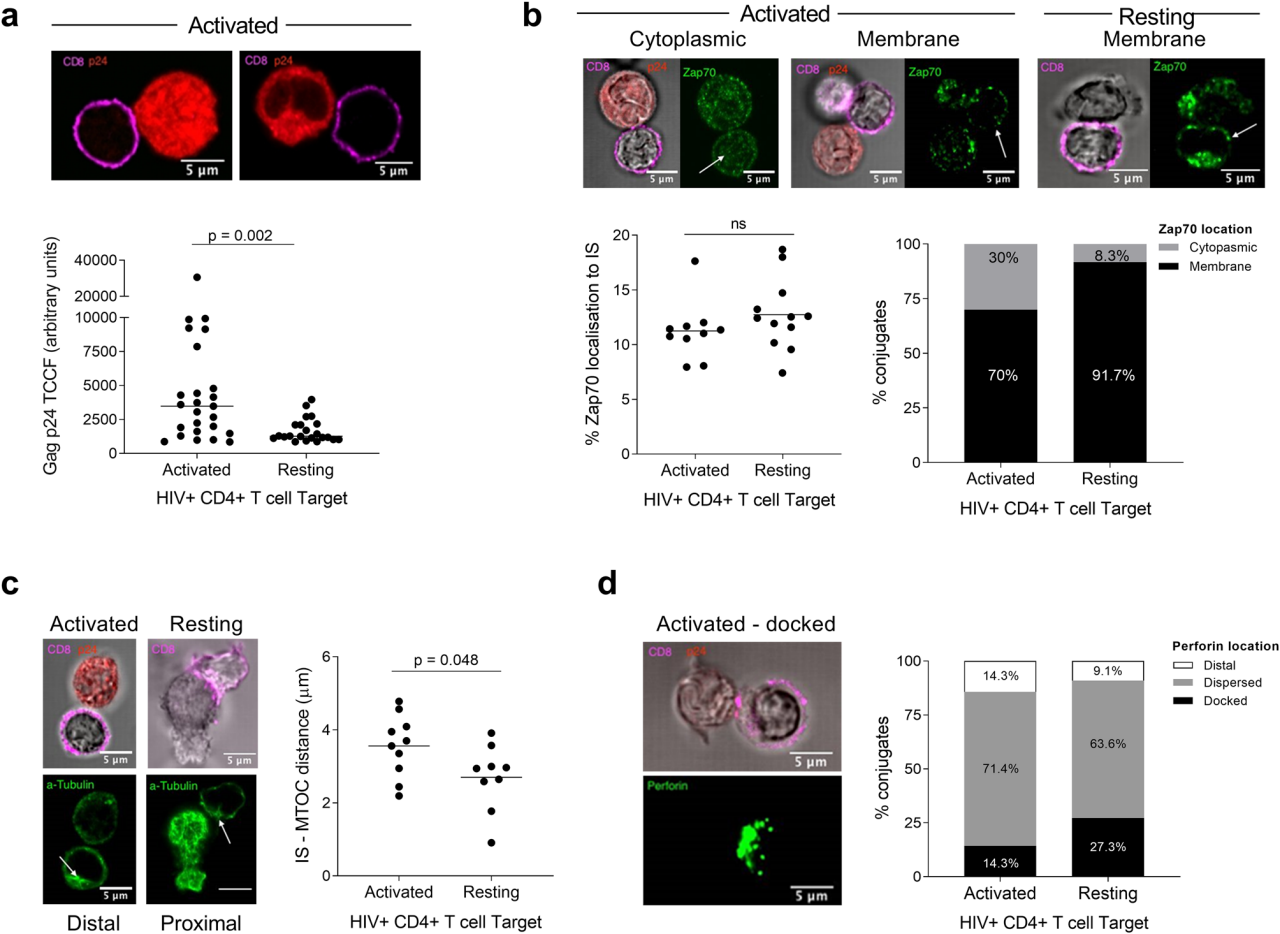
ImmTAV redirection of CD8^+^ T cells to HIV-infected primary CD4^+^ T cells. (**a**) Gag p24 total corrected cellular fluorescence (TCCF; as measured from confocal microscopy images) of activated or resting HIV-infected primary CD4^+^ T cells in conjugates with ImmTAV-redirected HIV-naïve donor CD8^+^ T cells (bottom); each dot represents a conjugate (n > 20/condition). Horizontal lines indicate median value. Groups were analysed by Mann Whitney test. Representative images of conjugates with activated infected CD4^+^ T cell targets shown as Gag expression in resting infected cells was not visible (top). (**b**) Confocal microscopy images of Zap70 localisation to the IS (examples of activated and resting, top), % Zap70 localisation at IS (bottom left) or cytoplasmic vs. membrane distribution (bottom right). (**c**) Confocal microscopy of α-tubulin (MTOC shown with white arrow; examples of activated and resting, left) and distance from MTOC to the synapse (µM, right). (**d**) Confocal microscopy of perforin localisation (docked example, left) and % of conjugates with distal, dispersed or docked perforin (right) in the CD8^+^ T cell. All synapse markers: at least 8 conjugates analysed per condition. Red = p24*, magenta = CD8 on DIC image, green = synapse molecule. For (**b**)–(**d**), horizontal lines indicate mean values. Groups were analysed by unpaired t test. *As p24 intensity in resting infected cells is very low and thus difficult to visualize, only DIC & CD8 signal images are shown.

The analysis of IS formed by CD8^+^ T cells from PLWH with peptide-pulsed targets suggested that perforin docking at the effector cell membrane was delayed relative to polarisation of the MTOC. We hypothesised that this delay could result in non-lytic synapses, which would be more apparent in the context of lower cognate peptide-HLA (pHLA) density than seen in the peptide-pulsed T2 used in Figs. 2 and 3. To develop a model system to explore this, we replaced peptide-pulsed T2 cells with more biologically relevant targets and initially assessed IS formation with CD8^+^ T cells from HIV-naïve HLA-A*02:01-positive donors. Primary resting (CD25^−^/CD69^−^/HLA-DR^−^) CD4^+^ T cells were spinoculated with HIV without prior activation and then cultured for 5 days. At this point they had low but detectable Gag expression while maintaining a resting phenotype and crucially, had downregulated CD4, indicating that viral integration and transcription of Nef had occurred (Supplementary Fig. S6)^12,36^. Gag expression was unaffected by the addition of a protease inhibitor (darunavir), which was included as an internal control, thus confirming that the resting infected cells were producing little if any infectious virus^37^. Immunofluorescence staining with a directly conjugated p24 antibody showed that Gag expression in resting infected CD4^+^ T cells was significantly lower than that of PHA-activated infected CD4^+^ T cells (Fig. 4a). These Gag^lo^ resting cells thus recapitulated the low antigen expression of HIV-infected CD4^+^ T cells circulating in ART-treated individuals.

We next assessed IS formation between ImmTAV-redirected HIV-naïve donor CD8^+^ T cells and either resting or activated HIV-infected CD4^+^ T cells following a 15-min co-culture. The percentage of IS localisation of Zap70 in CD8^+^ T cells was similar for activated and resting HIV-infected CD4^+^ T cells (11.3 ± 2.7% vs. 12.7% ± 3.2%; Fig. 4b, *left panel*). However, the percentage of conjugates with Zap70 localised at the cell membrane was higher in resting than activated cells (Fig. 4b, *right panel*). In addition, while MTOC polarisation and perforin docking were observed with both activated and resting infected CD4^+^ T cells, MTOC distance from the IS was shorter in CD8^+^ T cells forming conjugates with resting infected CD4^+^ T cells (activated vs. resting, mean µM (SD): 3.6 (0.8) vs. 2.7 (0.9), *p* = 0.048; Fig. 4c). Furthermore, there was a higher proportion of CD8^+^ T cells with perforin docked at the synapse with resting infected CD4^+^ T cells (activated vs. resting, % docked: 14.3% vs. 27.3%; Fig. 4d). These data indicated that the levels of Gag pHLA complexes presented on resting cells was sufficient to trigger IS formation by ImmTAV-redirected CD8^+^ T cells. Moreover, this process appeared to be faster in resting CD4^+^ T cells. However, the number of conjugates observed in both conditions was low (n = 9–12/condition).

**Figure 5:**
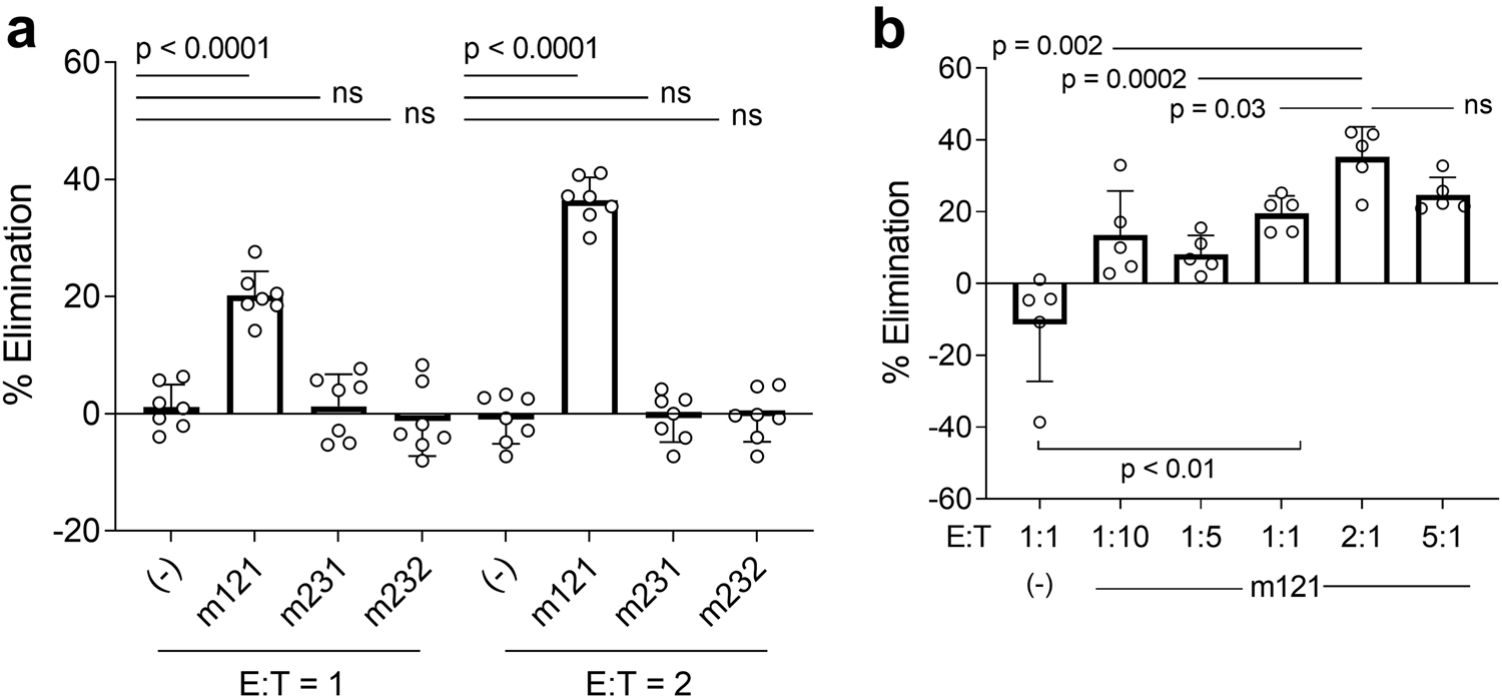
Resting infected CD4^+^ T cells are susceptible to ImmTAV-mediated killing. Resting infected CD4^+^ T cells from HIV-naïve donors (HD) were co-cultured with (**a**) autologous HD CD8^+^ T cells (E:T of 1:1 or 2:1) and m121 (0.5 nM), m231 (non-CD3 binding TCR fusion protein; 1 nM) or m232 (irrelevant TCR-anti-CD3 fusion protein; 1 nM) or (**b**) autologous HD CD8^+^ T cells at varying E:T ratios with 0.5 nM m121 for 48 h. The proportion of Gag^+^ cells remaining after ImmTAV exposure was used to determine the % elimination (normalised to % Gag^+^ cells in infected CD4^+^ T cells cultured alone). Horizontal lines indicate means. Groups were analysed by one-way ANOVA with Dunnett’s multiple comparisons test. Data shown are representative of two donors and four independent experiments.

We next sought to confirm that Gag^lo^ resting CD4^+^ T cells were susceptible to ImmTAV-mediated killing. Resting infected CD4^+^ T cells were cultured with HIV-naïve donor CD8^+^ T cells and m121 ImmTAV (0.5 nM). An irrelevant TCR/anti-CD3 fusion protein (m232) and a non-CD3 binding TCR fusion protein with the same TCR specificity as m121 (m231) were included as negative controls (both at 1 nM). At an effector to target ratio (E:T) of 1:1, the proportion of cells killed was 19% and this increased to 37% when the ratio was doubled (2:1), while neither of the control molecules was able to eliminate resting infected cells at either ratio, thus confirming the specificity of ImmTAV-redirected killing (Fig. 5a). Efficiency of killing also reached maximum at the 2:1 ratio as the addition of more effector cells (5:1) did not enhance the elimination of infected cells (Fig. 5b).

### ImmTAV redirection of bulk CD8^+^ T cells from PLWH overcomes the insensitivity of Gag^lo^ resting CD4^+^ T cells to autologous CTL

**Figure 6:**
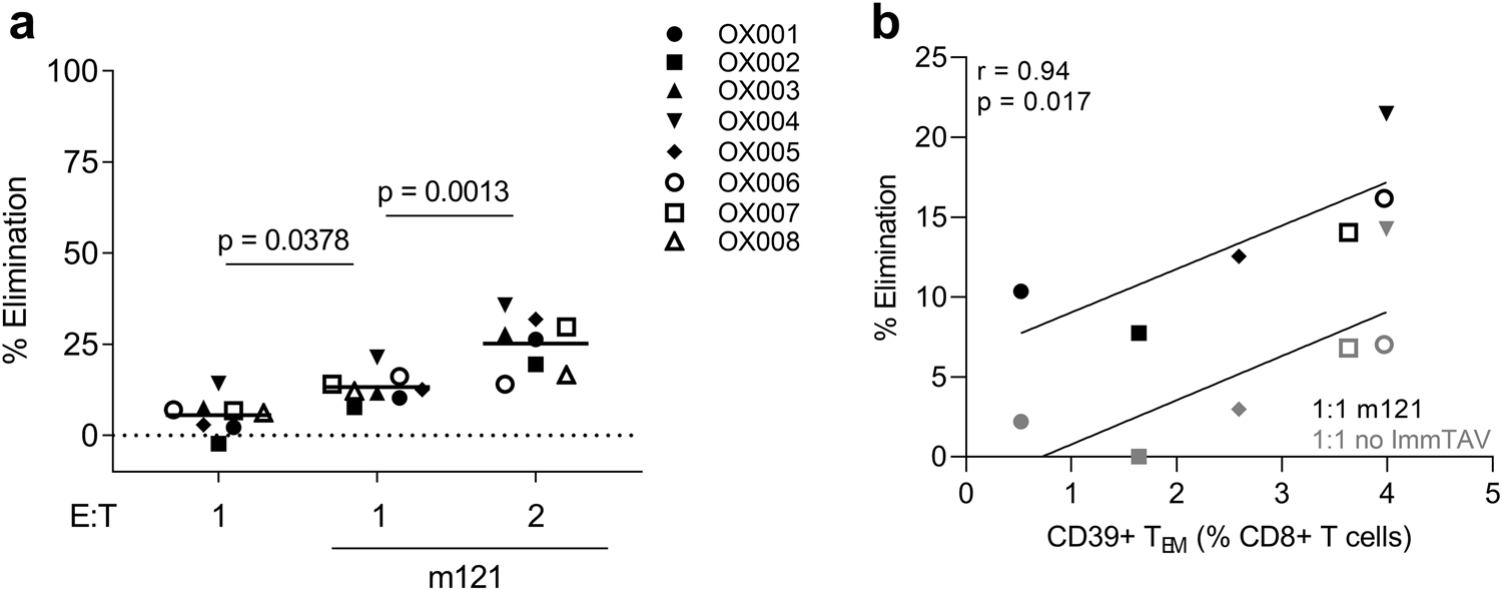
Elimination of resting infected CD4^+^ T cells by PLWH CD8^+^ T cells. Resting infected HD CD4^+^ T cells were co-cultured with CD8^+^ T cells from eight PLWH (E:T of 1:1 or 2:1) with and without m121 ImmTAV (0.5 nM) for 48 h. (**a**) Percent elimination was normalised to the percentage of Gag^+^ cells in infected CD4^+^ T cell cultures alone. For technical reasons, the no ImmTAV condition at an E:T ratio of 2:1 was not included. (**b**) The percent elimination data from (**a**) for six of the PLWH with phenotyping data was plotted against CD39 expression in the T_EM_ population with and without m121 ImmTAV. Mean values are indicated by horizontal bars. Groups were analysed by one-way ANOVA with Tukey’s multiple comparisons test or Spearman’s correlation.

As ImmTAV-redirected CD8^+^ T cells from PLWH were able to form IS with peptide-pulsed targets and to release granzymes (Figs. 1 and 3), we reasoned that they should also be capable of killing resting infected CD4^+^ T cells. Therefore, we cultured CD8^+^ T cells (E:T of 1:1 or 2:1) from eight PLWH with resting infected CD4^+^ T cells in the absence or presence of 0.5 nM m121 ImmTAV for 48 h. ImmTAV-redirected infected cell elimination was significantly greater than under conditions without the HIV ImmTAV, indicating that pre-existing HIV-specific CTL in these individuals were generally unable to mediate specific killing of Gag^lo^ targets (*p* = 0.0378; Fig. 6a). Furthermore, the ImmTAV-mediated effect was doubled at a 2:1 E:T ratio (*p* = 0.0013; mean ± SD 13.7% ± 4.1 and 26.8% ± 6.8 for E:T ratios of 1:1 and 2:1, respectively, and 5.6% ± 4.8 for CD8^+^ T cells without ImmTAV at an E:T ratio of 1:1). These data demonstrate that PLWH CD8^+^ T cells were able to mediate killing of Gag^lo^ targets over 48 h of culture. Finally, analysis of the six PLWH donors for whom CD8^+^ T cell phenotype was analysed in the same sample revealed a positive correlation between the frequency of CD39 + effector memory T cells determined ex vivo and the percentage elimination of resting infected CD4^+^ T cells in both the absence and presence of ImmTAV during the culture (E:T = 1:1, Spearman r = 0.94, *p* = 0.017; Fig. 6b).

## Discussion

A key assumption underpinning the development of bispecific biologics for chronic infections, including related molecules such as BiTEs and dual-affinity retargeting agents (DARTs), is that functionally intact polyclonal T cells can be recruited (reviewed in^38^). While numerous studies have reported the progressive loss of effector functions in HIV-specific CTL during the course of untreated infection that may not be restored by ART, the function of non-HIV-specific CD8^+^ T cells has not been extensively studied. Previous studies have reported that so-called chronic bystander viral infections could impair the differentiation of naïve CD8^+^ T cells into functional memory cells^1,39–42^. Given the high prevalence of ‘bystander’ infections such as CMV in PLWH, it was crucial to address this in order to assess the potential for bispecific retargeting agents to provide clinical benefit to PLWH.

A phenotypic analysis of polyclonal CD8^+^ T cells from PLWH who were durably suppressed on ART showed that they displayed several features of exhaustion that have been described primarily in virus-specific CD8^+^ T cells of viremic individuals. These included enrichment for TIGIT, Eomes and T-bet, with a skewing of the T-bet/Eomes ratio, and upregulation of CD39 in the effector memory subset^30–32,43,44^. Thus, our data indicate that such abnormalities can affect CD8^+^ T cells irrespective of their specificity and can persist during long-term ART. However, we also found that CD8^+^ T cells were capable of secreting granzyme B and perforin, consistent with intrinsic lytic potential, and were able to eliminate a minority of resting CD4^+^ T cells with low Gag expression when redirected by an HIV-specific ImmTAV, a potent TCR-anti-CD3 bispecific molecule. In a subset of individuals for whom phenotype and functionality were assessed simultaneously, the reduction in killing of these Gag^lo^ cells was proportional to the frequency of CD39^+^ effector memory T cells. Since CD39 and TIGIT upregulation are reported to be features of exhausted T cells, the detection of lytic activity in this context appears paradoxical. However, a recent report from Blazkova et al. noted that the frequency of TIGIT^+^ cells was positively correlated with the lytic capacity of CD8^+^ T cells from aviremic (ART-treated) individuals when redirected by an HIV-specific DART, calling into question whether TIGIT expression can be used to define a state of functional exhaustion^33^. Furthermore, CD39 upregulation could reflect recent T cell activation.

Nevertheless, we observed that PLWH CD8^+^ T cells eliminated a smaller percentage of Gag^lo^ targets than did CD8^+^ T cells from HIV-naïve donors. While we caution that these observations were made in separate experiments, given that the lytic activity of CD8^+^ T cells is dependent on the formation of stable and functional IS, we hypothesised that this could be related to the capacity of CD8^+^ T cells from PLWH to form functional IS. Using a co-culture system in which peptide-HLA density on the target cell could be controlled, we demonstrated that ImmTAV redirection of polyclonal CD8^+^ T cells from HIV-naïve donors towards peptide-pulsed T2 cells resulted in the mobilisation of Zap70, F-actin, MTOC and perforin within 15 min. By contrast, CD8^+^ T cells from PLWH showed desynchronisation of MTOC and perforin polarisation when redirected by the ImmTAV molecule. Polarisation of the MTOC and of lytic granules to the synapse were shown to be independently regulated according to the strength of TCR signals in a transgenic mouse model^45^. Our data suggest that signal transduction by CD8^+^ T cells from PLWH may be impaired, resulting in less efficient cytotoxicity despite high expression of lytic molecules.

Peptide-pulsed targets enabled a quantitative assessment of IS formation using primary CD8^+^ T cells as immune effectors, which was a key objective of this study. However, peptide-pulsed T2 cells are poorly representative of infected CD4^+^ cells that persist in ART-treated individuals. Resting HIV-infected primary CD4^+^ T cells were chosen for subsequent IS analysis, given that they express Gag proteins at low levels compared to activated infected CD4^+^ T cells, thus modelling the transcriptionally active component of HIV reservoirs in aviremic ART-treated individuals. In this experimental model we showed that HIV-naïve donor CD8^+^ T cells could form comparable IS with resting and activated infected CD4^+^ T cells. However, a low number of conjugates was observed, in comparison to the IS observed using peptide-pulsed targets. This therefore precluded extension of the IS analysis to PLWH CD8^+^ T cells under the same conditions. Despite this limitation, the observed reduction in resting HIV-infected cells by ImmTAV-redirected CD8^+^ T cells from PLWH in the infected cell elimination assay, provides supporting evidence for lytic IS formation with targets expressing cognate pHLA at low density. In addition, this cytotoxicity assay showed that resting infected CD4^+^ T cells were largely insensitive to pre-existing autologous HIV-specific CTL, which is consistent with previous studies in which models of non-productive infection were used to assess HIV-specific CTL potency in HIV controllers and chronic progressors^46,47^. This could be a consequence of low target expression in primary resting infected CD4^+^ T cells, functional exhaustion in autologous HIV-specific CTL, or a combination of the two.

In summary, we made use of a potent TCR-anti-CD3 bispecific molecule to demonstrate IS formation between primary HIV-infected CD4 + T cells and polyclonal CD8^+^ T cells from individuals with treated chronic HIV infection. We showed that the latter had phenotypic characteristics of exhaustion and formed poorly coordinated IS when compared to HIV-naïve donors. Nevertheless, redirection by ImmTAV molecules resulted in detectable killing of resting infected primary CD4^+^ T cells, thus overcoming a limitation of naturally primed HIV-specific CTL. The susceptibility of resting infected primary CD4^+^ T cells to ImmTAV-mediated killing supports further evaluation of these molecules for their potential to target the transcriptionally active component of HIV reservoirs independently of latency reversal. A final consideration is that earlier ART initiation might preserve intrinsic CD8^+^ T cell lytic capacity, since treatment during primary HIV infection reduces T cell activation and preserves specific antiviral functions^48,49^. While beyond the scope of this study, the potential for TIGIT blockade to enhance the effects of HIV ImmTAV redirection needs to be explored.

## Methods

### Cell lines and primary cells

T2 antigen presenting cells (174xCEM.T2) and Jurkat cells were obtained from American Type Culture Collection (ATCC) and were cultured in RPMI media supplemented with 10% foetal calf serum (FCS), 1% (v/v) penicillin/streptomycin and 2 mM L-glutamine (R10 media). HLA-A*02:01/β2M (A2B2M) was ectopically expressed in Jurkat cells by lentivirus transduction, with transductions confirmed by HLA-A2 staining (anti-HLA-A2 FITC, BioLegend, California, USA) and flow cytometry.

PBMC from HIV-naïve HLA-A*02:01-positive donors were isolated from buffy coats supplied by the NHS Blood Transfusion Service, Bristol, UK. PBMC from 29 HLA-A*02:01-positive subjects with chronic HIV infection (PLWH) receiving long-term ART for at least 12 months, with plasma HIV RNA < 50 copies/ml, and from HIV-naïve donors were acquired after obtaining ethical approval and written informed consent (Gene Therapy Advisory Committee, GTAC 165; Oxfordshire Research Ethics Committee, 10/H0604/95; South West—Central Bristol Research Ethics Committee, 16/SW/0331) (Supplementary Table S1). All methods using samples from human participants were performed in accordance with the relevant guidelines and regulations. CD4^+^ and CD8^+^ T cell subsets were isolated by magnetic bead selection or depletion following the manufacturer’s instructions (MACS, Miltenyi). In selected experiments, SL9-specific short-term CD8^+^ T cell lines were generated by stimulating PBMC from PLWH with SL9 peptide (4 μg/ml) followed by culture in RPMI media with IL-7 (25 ng/ml), IL-2 (1.8 × 10^3^ units/ml) and 10% human serum (H10 media) for 10 days. The SL9-specific CD8^+^ T cell population was measured by HLA-A*02:01-peptide dextramer staining as previously described (Immudex)^17^.

### Antibodies for immunofluorescence

HIV-infected cells were stained with the following primary antibodies: human anti-HIV-1 Gag p24 (37G12: Centre for AIDS Reagents, NIBSC, UK, supported by EURIPRED (EC FP7 INFRASTRUCTURES-2012 -INFRA-2012-1.1.5.: Grant Number 31266; Polymun) conjugated to Aberrior STAR 635P dye (Abberior GbmH) by NHS chemistry. Conjugation was performed by incubation of concentrated 37G12 (2–4 mg/ml), 0.2 M NaHCO_3_ (pH 8.3) and Aberrior STAR 635P NHS Ester (3:1 molar dye:antibody ratio) in PBS for 1 h at RT, followed by removal of unbound dye by filtration through Spin-X UF 500 50 k MWCO columns (Corning). Antibody concentration and degree of labelling was determined by NanoDrop. CD8^+^ T cells were identified using a mouse anti-human CD8α (clone 37,006, R&D Systems) or rabbit anti-human CD8α (Abcam). Immunological synapses were imaged using antibodies against perforin (clone dG9; BioLegend), α-tubulin (Abcam) and Zap70 (Abcam) or the Alexa Fluor 488 phalloidin stain (Molecular Probes). All unconjugated mouse and rabbit primary antibodies were labelled with a goat secondary antibody coupled to Alexa Fluor 488 or 568 (Life Technologies).

### Preparation of HIV-infected CD4^+^ T cells

CD8-depleted PBMC were rested for 2–24 h and stained with CD25-PE, CD69-PE and HLA-DR-PE antibodies (BD Biosciences). The activated cell fraction was removed by magnetic depletion using anti-PE microbeads (MACS, Miltenyi), leaving a resting cell (CD25^−^/CD69^−^/HLA-DR^−^) fraction that was 97% pure as confirmed by flow cytometry. Resting cell infection was achieved after culture in R10 media for 3 days using a previously described method: cells were spinoculated with HIV-1 IIIB (Centre for AIDS Reagents, National Institute for Biological Standards and Control; MOI = 0.01–0.1) at 2000 rpm for 2 h at 27 °C, washed with DNase I and cultured in R10 for a further 5 days before use^37^. An aliquot of 2 × 10^5^ cells was cultured with darunavir (10 μM) as a control to confirm absence of spread of infectious virus in resting cells. Activated cell infection was achieved by culturing CD8-depleted PBMC with PHA (5 μg/ml) for 3 days at 37 °C followed by spinoculation with HIV-1 IIIB (MOI = 0.01). After washing, cells were cultured in R10 supplemented with IL-2 (20 IU/ml) for 5 days.

### Preparation of cell conjugates for immunofluorescence microscopy

T2 cells (5 × 10^5^/slide) were pelleted and pulsed with SLYNTVATL (SL9) peptide (12.5 μM) in R0 for 5.5 h at 37 °C, washed and mixed with CD8^+^ T cells (1:1) and ImmTAV in R10 (up to 300 μl/well). Resuspended cells were added to prepared coverslips in a 24-well plate and allowed to settle for 5–30 min before fixation with 4% PFA. For analysis of primary CD4^+^ T cell/CD8^+^ T cell conjugates, HIV-1 infected CD4^+^ T cells were mixed with CD8^+^ T cells and ImmTAV at various effector/target ratios and slides were prepared as for T2 cells.

### Quantification of peptide-HLA complexes by microscopy

T2 cells pulsed with peptide as described above were stained and imaged for epitope quantification as previously described, with some modifications^17,50^. In short, pulsed or unpulsed cells were stained using biotinylated m121 TCR (50 nM) and CF640R-conjugated streptavidin (Biotium; 1 µg/ml) followed by Alexa Fluor 488 annexin V. Cells were fixed with 4% paraformaldehdye then transferred to poly-L-lysine coated glass-bottomed chambers. A Nikon ECLIPSE Ti2 microscope with an oil 100 × objective was used to first acquire tile images (phase-contrast and annexin V) to identify alive cells before Z-stack (0.8 µm apart) fluorescent images were taken to cover the entire 3D surface of the cell. The fluorescent spots, corresponding to TCR bound to pHLA, on each Z-stack for each cell were counted (at least 40 individual cells/condition) using Nikon NIS-Elements software.

### Staining for immunofluorescence microscopy

Cells were allowed to settle on fibronectin (5 μg/ml in PBS), or poly-L-lysine (0.01% w/v in H_2_O) coated glass coverslips for up to 30 min, fixed with 2–4% PFA and permeabilized with 0.1% Triton-X100 for 10 min. Cells were blocked with 2.5% goat serum/0.5% BSA in PBS for 45 min at room temperature (RT) then stained with primary antibodies for 1 h, followed by the secondary antibody for 1 h at RT. After washing, coverslips were sealed to glass slides and stored at 4 °C.

### Confocal microscopy image acquisition

Immunofluorescence images were acquired using either an LSM 510 or 880 inverted confocal microscope (Zeiss) with Zen software (v.2.1, Zeiss). A 63 × and 40 × plan-apochromat objective were used for immunological synapse analysis and conjugate quantification respectively. A differential interference contrast image (DIC) was acquired for assessment of cell morphology.

Slides were initially scanned for conjugates comprising a CD8^+^ cell and CD8^−^ cell to identify the correct cell pairing. For immunological synapse imaging, a single image in all colours (CD8 in 568 nm channel; synapse marker in 488 nm channel; where applicable, Gag p24 in 647 nm channel; DIC) was taken at the cell equator, followed by a Z-stack of the synapse marker channel (26 images, 0.37 Z-step μm). At least 10 conjugates per experimental condition were imaged. The number of T2-T cell conjugates were quantified by first randomly selecting regions containing T2 cells and then using the 40 × objective to acquire multiple overview images of the slide, until at least 50 target cells had been identified.

### Infected cell elimination assay

Resting infected CD4^+^ T cells (day 5 post-infection) were cultured with CD8^+^ T cells from HIV-naïve or PLWH at various effector/target ratios with or without ImmTAV molecules for 2 days. Infected cell frequencies were determined by intracellular Gag p24 staining and flow cytometry as described previously^17^.

### Granzyme B ELISpot assay

Granzyme B ELISpots were conducted according to the manufacturer’s instructions (BD Biosciences, cat# 552572). Briefly, SL9 peptide-pulsed (10 ng/ml) or unpulsed T2 cells (5 × 10^4^/well) were cultured with PBMCs (5 × 10^4^/well) from HIV-naïve donor or PLWH, together with m121 ImmTAV (0.5 nM) or control TCR (m231, 1 nM) in R10 medium in pre-coated ELISpot plates for 48 h. PHA stimulation was used as a positive control for cell viability. Granzyme B was detected using a biotinylated anti-granzyme B antibody followed by addition of streptavidin-HRP and enzyme substrate. Spots were counted using ImmunoSpot software (CTL).

### Flow cytometric analysis

Cells were washed with PBS and stained first with LIVE/DEAD stain followed by combinations of cell surface markers, including: CD3 APC-Fire750, CD4 PE-CF594, CD8 PerCP-Cy5.5, CD45RA PE-Cy7, CD27 APC, PD-1 PE, CD39 BB515, CCR7 BV421 (all BioLegend), TIGIT-BV421, CD57-BB515 (BD Biosciences). For staining of transcription factors surface Foxp3 Transcription Factor Staining Buffer (Invitrogen) was used, according to manufacturer’s instructions, to fix and permeabilize cells before staining with T-bet-BV786 (BD Biosciences) and Eomes-PE-eFluor 610 (Invitrogen). Samples were fixed with 2% PFA, acquired on either a CyAn (Beckman Coulter) or LSR Fortessa (BD Biosciences) flow cytometer and analysed using FlowJo v9.9.6 software (FlowJo, Ashland, OR, USA).

### Production of ImmTAV molecules

TCR isolation and engineering to produce ImmTAV reagents have been described previously^15,51^.

### Analysis of confocal images

All analysis of microscopy images was done using ImageJ (NIH). To determine p24 expression of CD8- cells the total corrected cellular fluorescence (TCCF) was calculated using the DIC image to outline the cell of interest and the p24 channel to take IntDen measurements (TCCF = IntDen_cell_ − (Area_cell_ * MFI_background_)). The mean TCCF(p24) of the uninfected cells was used as a cut-off for the detection of p24 in the target cells; conjugates with CD8-negative cells with TCCF(p24) values below the mean were excluded from further analysis.

For Zap70 cell distribution, conjugates were defined as having Zap70 localized to the membrane or the cytoplasm based on the signal distribution in acquired confocal images. Zap70 analysis of the % protein at the contact (% of total) was calculated using the IntDen measurements from a summed Z stack in that channel (% at contact = (IntDen_synapse_/IntDen_whole cell_)*100). The distance from the MTOC to the centre of the IS was measured using the α-tubulin images. Perforin was categorized as being either distal (far side from IS), dispersed (spread through the cell) or docked (next to the IS).

To quantify the number of T2-T cell conjugates that formed, low power images (CD8 and DIC channels) were taken across the slide. The total number of T2s (at least n = 50) and the number in conjugates with CD8^+^ T cells was counted (blinded to the condition) and was compared across conditions.

### Live cell imaging

Jurkat cells were infected with HIV-IIIB and co-cultured with HIV-naïve donor CD8^+^ T cells (E:T 1:1) and 5 nM m121 ImmTAV in a chambered cover glass. The cover glass was added to the microscope stage (in Cat III containment) and after 5 min, to allow for temperature calibration, the slide was scanned for a suitable field of view. Images were taken every two seconds for 30 min and the images were stitched together to form the movie.

### Statistical analysis

Statistical analysis was performed using GraphPad Prism software (version 6.0 or later). Where a normal distribution was assumed, data were analysed with parametric tests ((Repeated Measures one-way ANOVA (with or without Dunnett’s, Tukey’s or Sidak’s multiple comparisons tests—adjusted *P* values shown), or t tests); data with skewed distribution were analysed with non-parametric tests (Mann–Whitney tests or Kruskal–Wallis tests).

## Supplementary Information

Supplementary Video 1.

Supplementary Information 1.

## Data Availability

The datasets generated and/or analysed during the current study are not publicly available as the data exists across multiple institutional servers but are available from the corresponding author on reasonable request.
