# Supplementary material for: Immune mobilising T cell receptors redirect polyclonal CD8+ T cells in chronic HIV infection to form immunological synapses
Source: Sci Rep. 2022 Nov 1;12:18366. doi: 10.1038/s41598-022-23228-3 (PMC9626491; doi:10.1038/s41598-022-23228-3)
Supplement: Supplementary file 2 — Supplementary Information 1. [file 41598_2022_23228_MOESM2_ESM.pdf]

Immune mobilising T cell receptors redirect polyclonal CD8$^+$ T cells in chronic HIV infection to form immunological synapses

Authors: Zoë Wallace[1,2,3*], Jakub Kopycinski[1], Hongbing Yang[1,2], Michelle L. McCully[3], Christian Eggeling[4,5], Jakub Chojnacki[4], Lucy Dorrell[1,2,3]

Supplementary Information

Supplementary Table S1. Percentage reduction in Gag$^+$ cells by ImmTAV-redirected CD8$^+$ T cells*

	**[ImmTAV]**	**m121**	**m121**	**m134**	**m134**
**Effector source**		HIV+	HIV-	HIV+	HIV-
**E:T = 1:1**	10 nM	60% (20%)	87% (6%)	72% (14%)	82% (11%)
**E:T = 1:10**	10 pM	55% (17%)	63% (23%)	55% (15%)	71% (18%)

*Previously published in (Yang et al., 2016), indicate mean (SD).

Supplementary Table S2. PLWH cohort characteristics

Donor ID	Gender	Age	ART duration (years)	$CD4^+$ T cell count (cells/μl)
**OX006**	F	43	4.5	680
**OX007**	M	55	1	640
**OX004**	M	29	2	400
**OX003**	M	46	5	610
**OX002**	M	44	15	720
**OX001**	M	52	12	760
**OX005**	M	33	2	390
**OX009**	M	28	>1*	N/A
**OX010**	M	45	2	370
**OX011**	F	41	6	870
**OX012**	M	46	4	690
**OX013**	F	33	3	400
**OX014**	M	44	4.5	490
**OX015**	F	44	9	990
**OX016**	M	46	>1*	360
**OX017**	F	46	2	430
**OX018**	M	40	12	1060
**OX019**	M	46	2	730
**OX020**	M	46	2.5	390
**OX021**	M	55	12	2010
**OX022**	M	50	5.5	560
**OX023**	F	37	4	440
**OX024**	M	39	3	450
**OX025**	M	48	2	630
**OX026**	M	43	1.5	930
**OX027**	M	60	1	360
**OX028**	F	27	1.5	1270
**OX029**	M	N/A	7.5	N/A
**OX008**	F	33	4	720

***** On ART for at least 1 year but exact duration not known

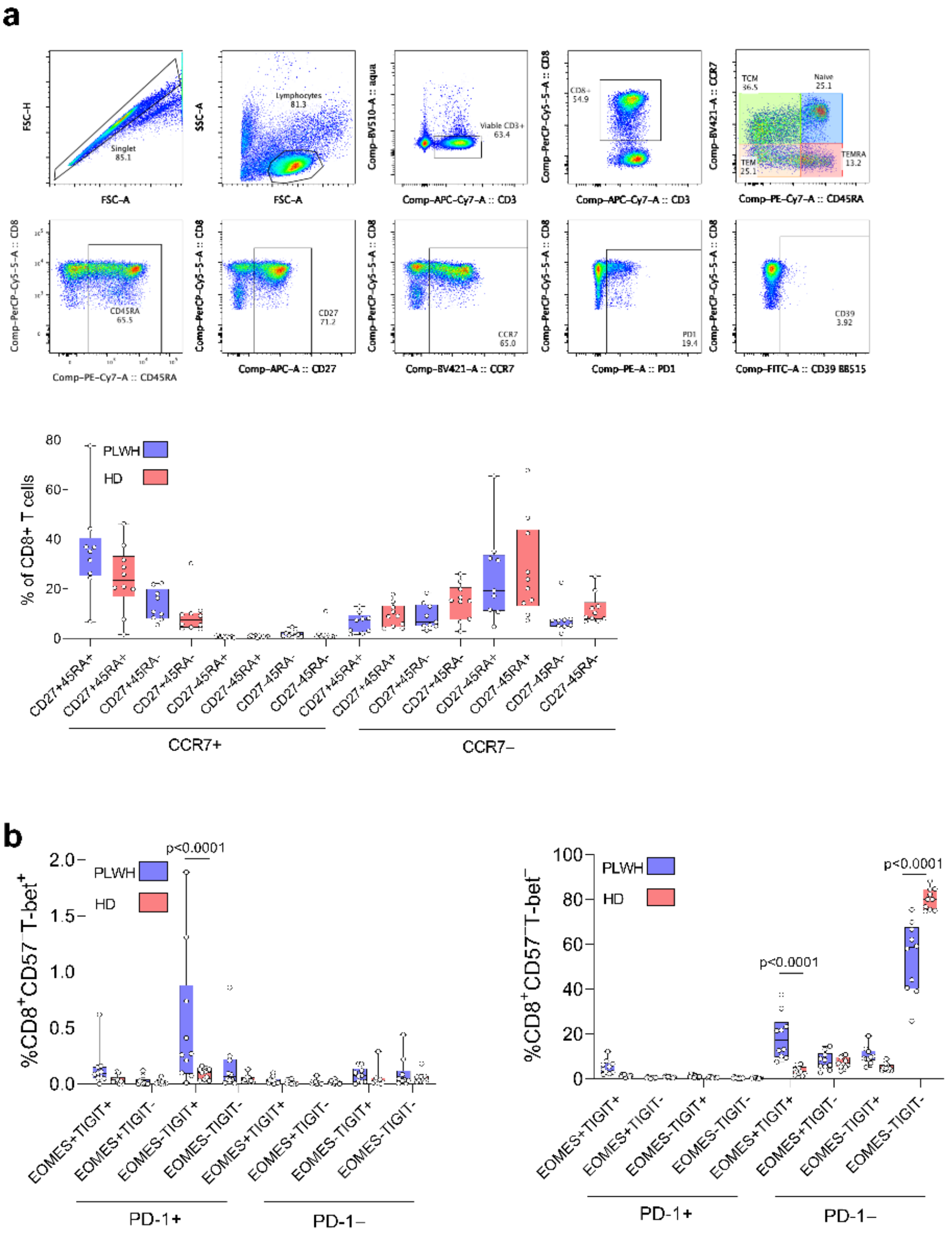

Supplementary Figure S1. Phenotype of PLWH and HD T cells.

CD8$^+$ T cells from nine PLWH (blue) and ten HIV-naïve donors (red) were analysed for expression of **(A)** CD45RA, CCR7 and CD27 (effector memory or terminally differentiated populations - gating shown: top row shows gating for single, lymphocyte, alive CD3+CD8+ T cells and memory populations (CD45RA/CCR7); bottom row shows gating for CD45RA, CD27, CCR7, PD-1 and CD39 on CD8+ T cells) with no significant difference between PLWH and HD. A second set of nine PLWH donors (blue) and ten HIV-naïve donors (red) were analysed for expression of **(B)** CD57, Eomes, PD-1, T-bet and TIGIT (CD57$^-$ population shown). Groups were analysed by 2-way ANOVA with Sidak's multiple comparisons. See Supplementary Table 2 for PLWH details.

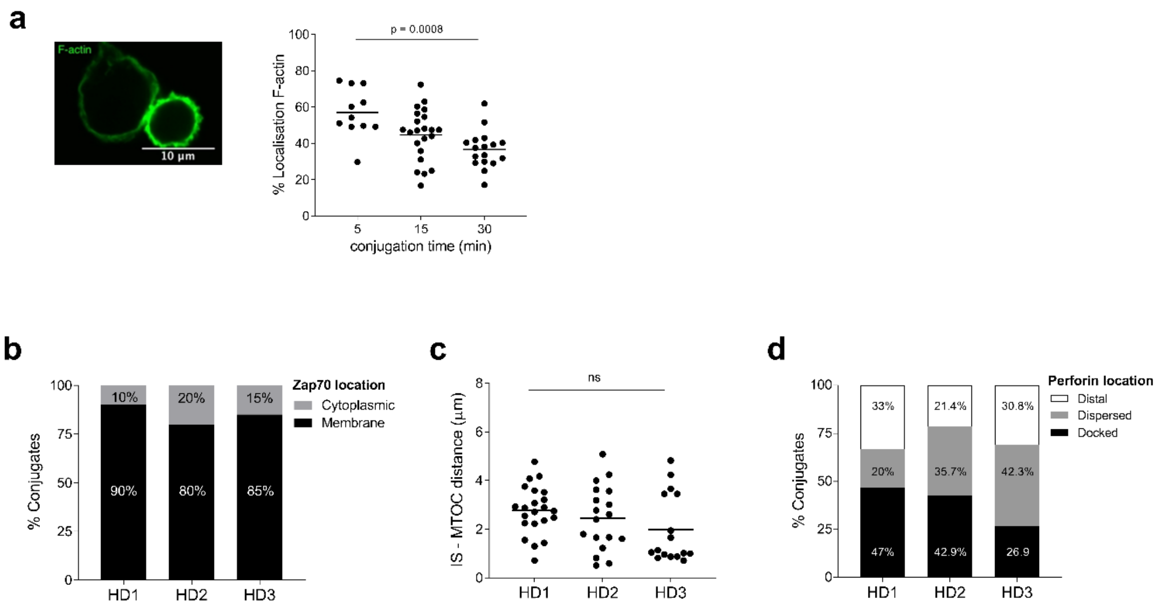

Supplementary Figure S2. Reproducibility of immune synapse formation with CD8^{+} T cells from three HIV-naïve donors (HD).

(A) Confocal microscopy image of bright F-actin expression in a conjugate formed between a HD CD8^{+} T cell/SL9-pulsed T2 cell and % localisation of F-actin at the IS over time (mean shown). Similar distribution of **(B)** Zap70, **(C)** MTOC and **(D)** perforin in CD8^{+} T cells from three HD when cultured with SL9-pulsed T2 cells at a ratio of 1:1 in the presence of ImmTAV (0.5 nM).

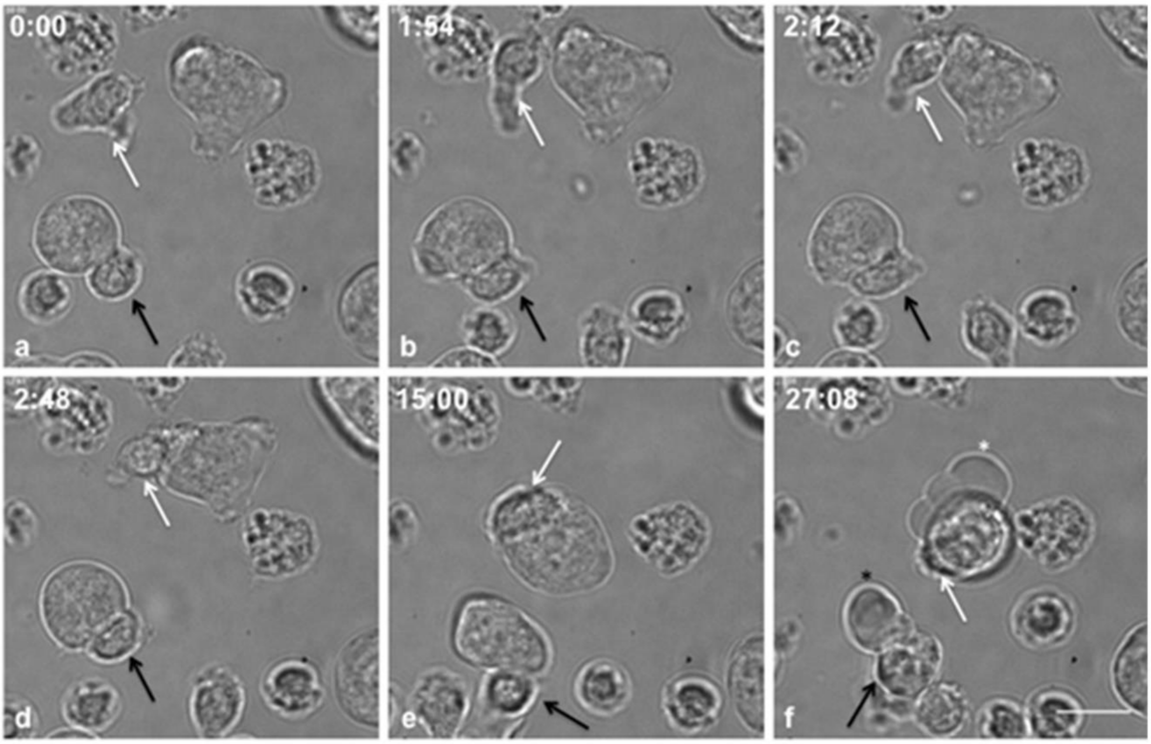

Supplementary Figure S3 and Video S1. Time-lapse microscopy of ImmTAV-redirected CD8$^+$ T cell killing of virus-infected CD4$^+$ T cells.

HIV-infected, HLA-A2 transduced Jurkat T cells were co-cultured with HD CD8$^+$ T cells (E:T of 1:1 and m121 ImmTAV at 5 nM). Timestamp on each image is in relation to the start of imaging at 0:00, 15 minutes after co-culture began. Images show stages of synapse formation for top conjugate (white arrow marking CD8$^+$ T cell) including (a) the migration of the CD8$^+$ T cell, (b) the CD8$^+$ T cell scanning the target, (c) the CD8$^+$ T cell making contact with the target, (d) the formation of a tight synapse, (e) the CD8$^+$ T cell roaming around the target and (f) the blebbing of the cell membrane of the target (*). Second conjugate already formed at 0:00 marked in black. Scale bar represents 10 μm. See Video S1.

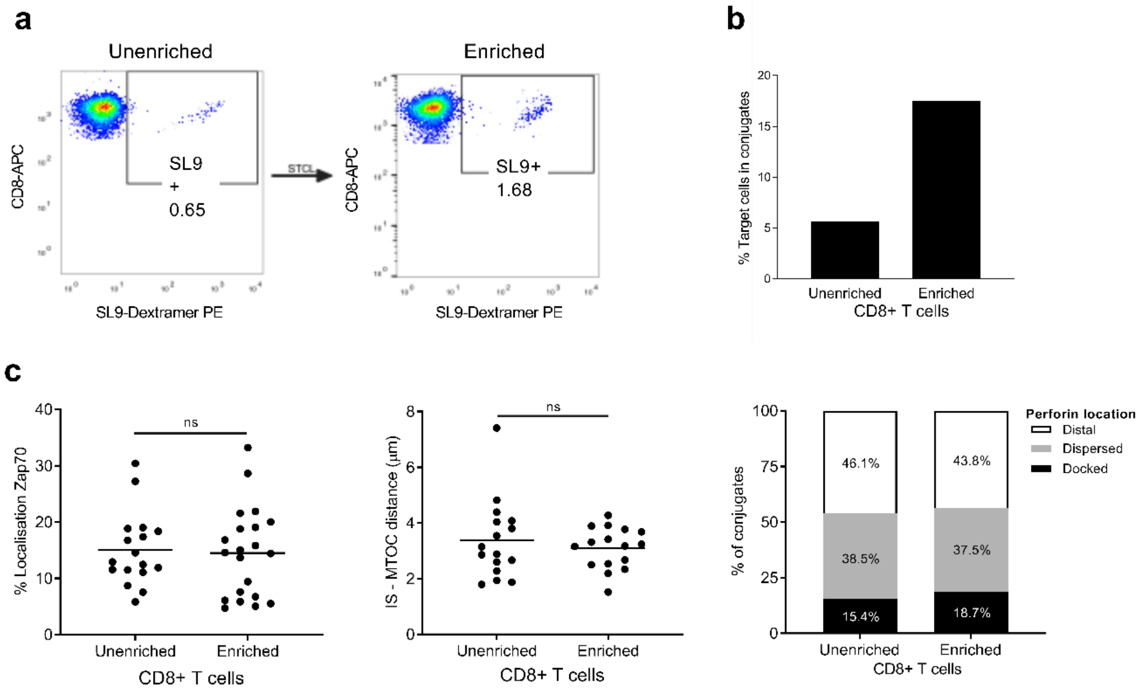

Supplementary Figure S4. Comparison of synapses formed with unenriched and enriched SLY-specific CD8$^+$ T cells.

Enrichment of SL9-specific CD8$^+$ T cells from PLWH donors as seen **(A)** by flow cytometry staining with an SLY-dextramer **(B)** increases the percentage of target cells in conjugates with SL9-pulsed T2s but does not affect **(C)** percentage localisation of Zap70 to the IS, localisation of the MTOC (µm from synapse) or perforin distribution (% of conjugates with perforin docked vs. dispersed vs. distal to the synapse). One donor shown (each dot represents one T2/CD8$^+$ T cell conjugate); data were analysed by unpaired t test.

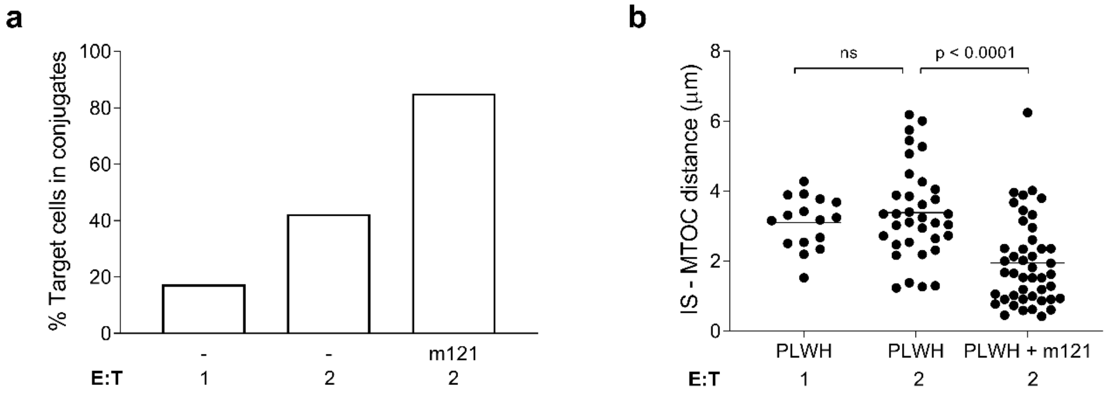

Supplementary Figure S5. Effect of E:T ratio and ImmTAV on conjugate formation. Increasing the E:T or addition of ImmTAV to co-cultures of PLWH donor $CD8^+$ T cells and SL9-pulsed T2s **(A)** increases the percentage of target cells in conjugates but **(B)** only addition of ImmTAV impacts the distance of the MTOC from the IS (µm). One donor shown (each dot represents one T2-$CD8^+$ T cell conjugate); data were analysed by one-way ANOVA with multiple comparisons.

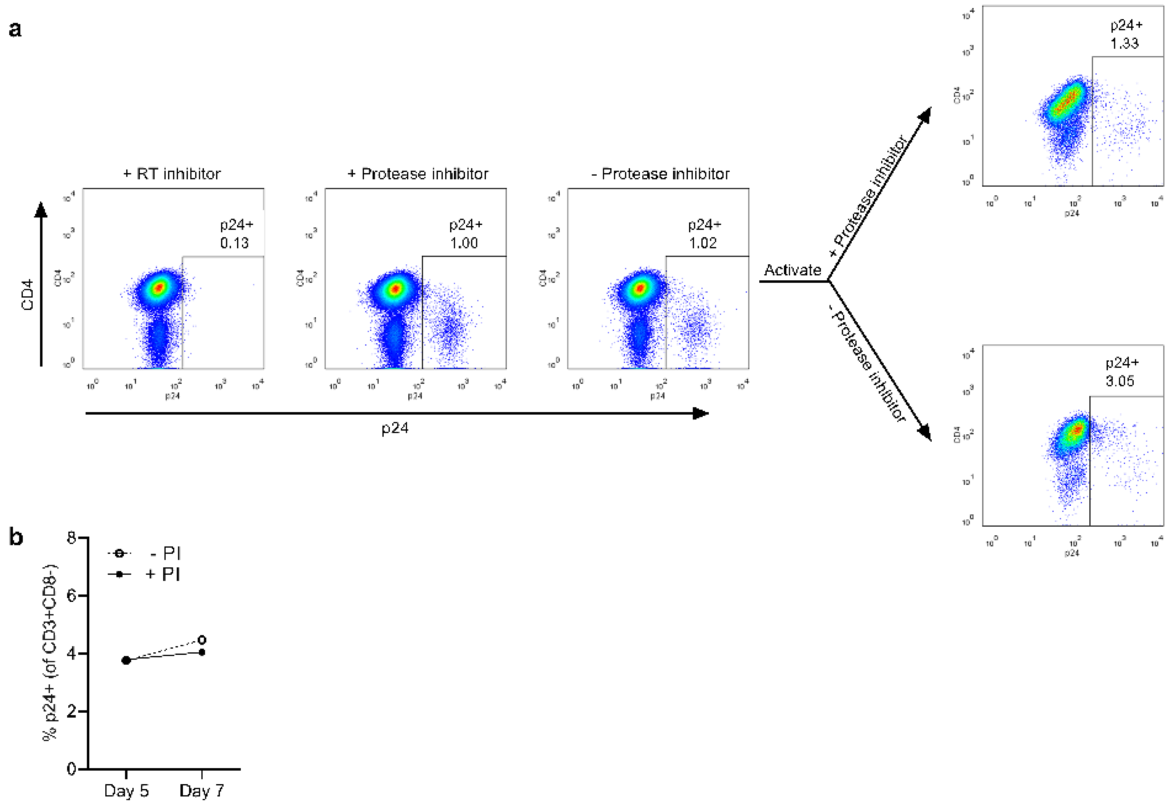

Supplementary Figure S6. Downregulation of CD4 in resting infected cells.

(A) Resting infected CD4^{+} T cells show downregulation of CD4 (y-axis) and expression of Gag p24 (x-axis) but do not show spreading infection (no difference in p24 expression when cultured +/- protease inhibitor (PI) until activated with PHA) (B) through the 7-day assay duration.
